# Cembranoids with 3,14-Ether Linkage and a Secocembrane with Bistetrahydrofuran from the Dongsha Atoll Soft Coral *Lobophytum* sp.

**DOI:** 10.3390/md9071243

**Published:** 2011-07-12

**Authors:** Mohamed Elamir F. Hegazy, Jui-Hsin Su, Ping-Jyun Sung, Jyh-Horng Sheu

**Affiliations:** 1 Department of Marine Biotechnology and Resources, National Sun Yat-sen University, Kaohsiung 804, Taiwan; E-Mail: elamir77@live.com; 2 National Museum of Marine Biology & Aquarium, Pingtung 944, Taiwan; E-Mails: x2219@nmmba.gov.tw (J.-H.S.); pjsung@nmmba.gov.tw (P.-J.S.); 3 Graduate Institute of Marine Biotechnology, National Dong Hwa University, Pingtung 944, Taiwan; 4 Division of Marine Biotechnology, Asia-Pacific Ocean Research Center, National Sun Yat-sen University, Kaohsiung 804, Taiwan

**Keywords:** soft coral, secocembrane, *Lobophytum*, tetrahydrofuran

## Abstract

Four new cembranoids, lobophylins A–D (**1**–**4**), and one novel secocembrane, lobophylin E (**5**) were isolated from a soft coral *Lobophytum* sp. The structures of new metabolites were elucidated on the basis of extensive spectroscopic methods. Among these metabolites, **1**–**4** are rarely found cembranoids possessing a tetrahydrofuran moiety with a 3,14-ether linkage. In addition, **5** is the first secocembrane possessing two tetrahydrofuran moieties with 3,14- and 4,7-ether linkages.

## 1. Introduction

**Chart 1:**
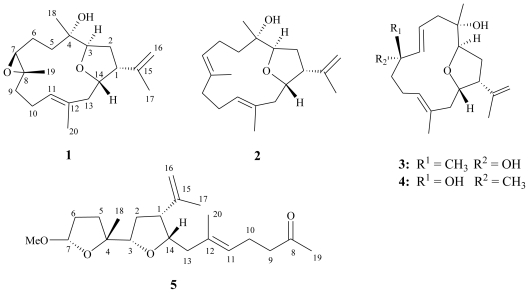
Structures of metabolites **1**–**5**.

Soft corals have proven to be important sources of secondary metabolites with interesting biological activities [1]. In the investigation of the secondary metabolites from soft corals in Taiwan waters, a series of bioactive cembranoids have been isolated from octocorals (Alcyonaceae) belonging to the genera *Sinularia* [2–7], *Lobophytum* [8–10], *Sarcophyton* [11–16] and *Pachyclavularia* [17,18]. Some of these metabolites have been shown to exhibit significant cytotoxic activity against the growth of various cancer cell lines [10,15–17], and/or anti-inflammatory activity [3,6,8,10,15,16]. Our previous chemical investigation on Dongha Atoll soft coral *Lobophytum sarcophytoides* has led to the isolation of bioactive cembranoids [19]. In our continuing search for bioactive metabolites from Dongsha Atoll soft corals of the genus *Lobophytum*, we investigated the chemical constituents of *Lobophytum* sp. and succeeded in the isolation of four new cembranoidal lobophylins A–D (**1**–**4**) and a novel secocembrane, lobophylin E (**5**) (Chart 1). The structures of these compounds have been established by extensive spectroscopic analysis. The cytotoxicity of compounds **1**–**5** against four human cancer cell lines was investigated, however, none of these was found to possess useful biological activity.

## 2. Results and Discussion

**Figure 1:**
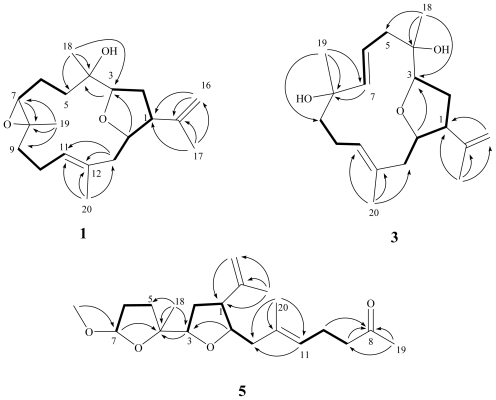
Selected ^1^H-^1^H COSY ( 
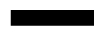
) and HMBC (→) correlations of **1**, **3** and **5**.

The new metabolite lobophylin A (**1**) exhibited a protonated molecule peak in the HRESIMS at *m/z* 343.2251 [M + Na]^+^, establishing the molecular formula C_20_H_32_O_3_ and five degrees of unsaturation. The IR spectrum suggested the presence of hydroxy group (ν_max_ 3460 cm^−1^) in **1**. The ^13^C NMR spectrum of **1** measured in CDCl_3_ (Table 1) showed the presence of twenty carbon signals, which were assigned by the assistance of DEPT spectrum to four methyls, six sp^3^ methylenes, one sp^2^ methylene, four sp^3^ methines (including three oxymethines), one sp^2^ methine, and two sp^3^ quaternary and two sp^2^ quaternary carbons. From the ^1^H NMR spectroscopic data of **1** (Table 2), the presence of two hydroxy protons resonating at δ 3.98 (dd, *J* = 9.6, 4.4 Hz) and 4.37 (ddd, *J* = 12.0, 3.6, 3.6 Hz) were observed. Moreover, the ^1^H NMR spectrum revealed the presence of two olefinic methylene protons at δ 4.87 (d, *J* = 1.6 Hz) and 4.81 (s) and one olefinic methine proton at δ 5.09 (t, *J* = 6.8 Hz). A proton signal appearing at δ 3.27 (^1^H, d, *J* = 6.8 Hz) and correlating with a carbon signal at δ 64.7 in the HMQC spectrum was due to the proton of the trisubstituted epoxide. The planar structure and all of the assignments of ^1^H and ^13^C NMR data of **1** were determined by the assistance of 2D NMR studies, including ^1^H-^1^H COSY and HMBC experiments (Figure 1). ^1^H-^1^H COSY spectrum revealed proton sequences from H-1 to H-3 and H-13 to H-1; H_2_-5 to H-7; H_2_-9 to H-11, as shown by the bold lines in Figure 1. Key HMBC correlations of H-3 to C-4; H-7 to C-8; H_2_-13 to C-11 and C-12; H_2_-16 to C-1 and C-15; H_3_-17 to C-1, C-15 and C-16; H_3_-18 to C-3, C-4 and C-5; H_3_-19 to C-7, C-8 and C-9; and H_3_-20 to C-11, C-12 and C-13, permitted the connection of the carbon skeleton. Furthermore, the HMBC cross-peak from H-14 to C-3 suggested that C-3 and C-14 were linked through an oxygen to form a tetrahydrofuran ring. Thus, **1** was revealed as a cembranoid possessing a 3,14-ether linked tetrahydrofuran ring, on the basis of the above analysis.

**Figure 2:**
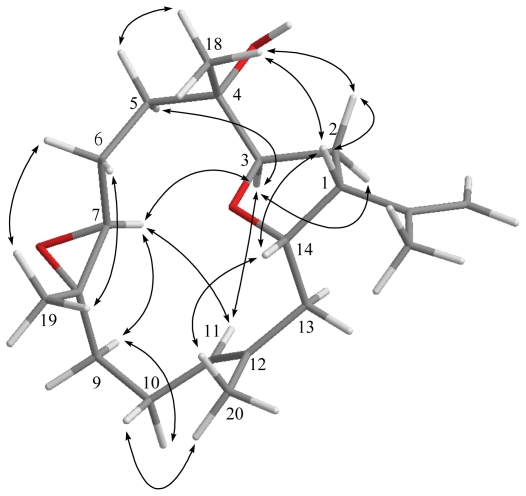
Computer-generated model for **1** using MM2 force field calculations and key NOE correlations.

The relative configuration of **1** elucidated mainly by NOESY spectrum was compatible with that of **1** offered by using the MM2 force field calculations which suggested the most stable conformations as shown in Figure 2. In the NOESY spectrum, it was found that H-1 (δ 2.77, dt, *J* = 8.8, 8.0 Hz) showed NOE interactions with H-14 and H_3_-18 (δ 1.15, s); therefore, assuming the β-orientation of H-1, H-14 and H_3_-18 should also be positioned on the β face. One of the methylene protons at C-2 (δ 1.92) exhibited NOE correlations with H-1 and was characterized as H-2β, while the other (δ 2.16) was assigned as H-2α. NOE correlations observed between H-2α and H-3 (δ 3.98, dd, *J* = 9.6, 4.4 Hz), and H-3 and H-7 (δ 3.27, d, *J* = 6.8 Hz), reflected the α-orientations of both protons H-3 and H-7. Also, H_3_-19 was found to interact with H_2_-6, but not with H-7, revealing the *trans* geometry of the trisubstituted epoxide. Furthermore, the NOE correlations observed H_3_-20 and H-10 (δ 2.21), but not with H-11, reflected the *E* geometry of double bond at C-11. On the basis of the above findings and other detailed NOE correlations (Figure 2), the relative structure of **1** was determined.

HRESIMS analysis of lobophylin B (**2**) provided a molecular formula of C_20_H_32_O_2_ ([M + Na]^+^ *m/z* 327.2301). The ^1^H and ^13^C NMR spectroscopic data of **2** were very close to those of **1** (Tables 1 and 2), except for the replacement of the two carbon signals of the epoxide moiety in **1** by the signals of a trisubstituted double bond in **2** (δ 126.6, CH, C-7 and 132.8, C, C-8). This double bond was positioned at C-7/C-8 due to the ^1^H-^1^H COSY correlation found between the H-6 and H-7, the HMBC correlations observed from the olefinic methyl protons at δ 1.65 (3H, s) to C-7, C-8 and C-9. Furthermore, the *E* geometry of the 7,8-double bond was deduced from the NOE correlation of H_3_-19 with H_2_-6 and not with H-7. Thus, the structure of **2** was determined unambiguously. Literature review revealed a known compound similar to compound **2** but possessing a rare 3,13-bridged tetrahydropyran ring [20].

**Figure 3:**
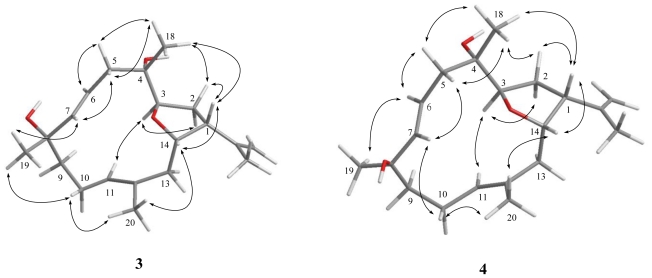
Computer-generated model for **3** and **4** using MM2 force field calculations and key NOE correlations.

Lobophylin C (**3**) showed a protonated molecule peak [M + Na]^+^ at *m/z* 343.2248 in the HRESIMS, corresponding to the molecular formula C_20_H_32_O_3_ and five degrees of unsaturation. The IR spectrum showed the presence of hydroxy (3377 cm ^−1^ ) group. ^1^H and ^13^C NMR spectroscopic data (Tables 1 and 2) of **3** showed the structural unit of a 3,14-oxa-bridged tetrahydrofuran, too. ^1^H-^1^H COSY and HMBC (Figure 1) further revealed that **3** possesses a 1,2-disubstituted double bond (δ 118.9 and 142.7, each CH) at C-6 and C-7 and a quaternary oxycarbon at C-8 (δ 73.6, C). On the basis of the above observations, and by the assistance of additional 2D NMR (^1^H-^1^H COSY and HMBC) correlations, it was possible to establish the planar structure of **3** as illustrated in Figure 1. The relative configurations of the five chiral centers at C-1, C-3, C-4, C-8 and C-14 in **3** were thus determined on the basis of NOE correlations (Figure 3). By careful inspection on the NOESY spectrum of **3**, it was found that one proton (δ 2.40) of H_2_-5 showed NOE interaction with both H_3_-18 and H-7, and H-7 was NOE correlated with H_3_-19. Therefore, H_3_-18 and H_3_-19 are situated on the same β-face. Furthermore, NOESY spectrum showed correlation of H_3_-20 with one proton (δ 2.19) of CH_2_-10, but not with H-11, revealing the *E*-configurations of the 11,12-trisubstituted double bond. The above finding, together with *J* values for both H-6 (15.2 Hz) and H-7 (15.6 Hz), confirmed the *E*-configuration of the 6,7-double bond. Further NOE analysis revealed that **3** possessed the same configurations at C-1, C-3, C-4 and C-14, as in compound **1** (Figure 3). Based on the above results, the structure of **3** was established.

The HRESIMS spectrum of lobophylin D (**4**) showed a molecular formula of C_20_H_32_O_3_, the same as that of **3**. By analysis 2D NMR spectra, including ^1^H-^1^H COSY, HMQC and HMBC, **4** was shown to possess the same molecular framework as that of **3**. Furthermore, it was found that the NMR data of **4** were very similar to those of **3** (Tables 1 and 2), revealing that **4** might be an isomer of **3**. However, the significant downfield shift at C-6 (Δδ_C_ +2.9 ppm) and the upfield shift at C-7 (Δδ_C_ −1.2 ppm), C-8 (Δδ_C_ −1.0 ppm) and C-19 (Δδ_C_ −1.3 ppm), relative to those of **3** (Table 2), suggested that **4** might be the C-8 epimer of **3**. From NOESY spectrum, it was found that one proton (δ 2.56, m) of H_2_-10 of **4** showed NOE correlations with H-7 (δ 5.75, d, *J* = 15.5 Hz) and H_3_-20 (δ 1.70, s), while H-6 (5.51, ddd, *J* = 15.5, 10.0, 5.0 Hz) was NOE correlated with H_3_-19 (δ 1.37, s) (Figure 3). Therefore, both H-7 and H_3_-20 are situated on the β-face, and in contrast, H-6 and H_3_-19 should be positioned on the α-face. This inferred the R* configuration at C-8. Further analysis of other NOE interactions revealed that **4** possessed the same relative configurations at C-1, C-3, C-4 and C-14 as those of **3** (Figure 3). Therefore, **4** was found to be the C-8 epimer of **3**.

**Figure 4:**
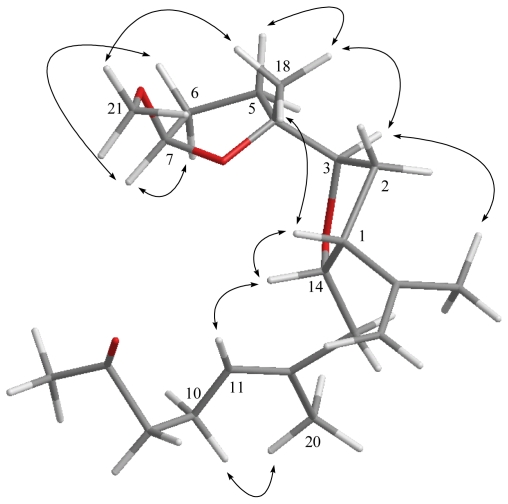
Computer-generated model for **5** using MM2 force field calculations and key NOE correlations.

Lobophylin E (**5**) was assigned a molecular formula of C_21_H_34_O_4_, according to the HRESIMS and NMR spectroscopic data (Tables 1 and 2). The IR absorption band at 3444 cm^−1^ revealed the presence of hydroxy group. By the analysis of ^13^C and DEPT spectroscopic data, the carbons signals were assigned into five methyls (including one methoxy methyl resonating at δ_C_ 54.3), six sp^3^ methylenes, one sp^2^ methylene, four sp^3^ methines (including two monooxygenated carbons resonating at δ_C_ 82.2 and 80.3 and an acetal carbon resonating at δ_C_ 105.6), one sp^2^ methine, one sp^3^ quaternary carbons and three sp^2^ quaternary carbons (including a normal ketone resonating at δ_C_ 208.9). From the ^1^H-^1^H COSY spectrum of **5**, it was possible to identify three different structure units, which were assembled with the assistance of an HMBC experiment. Key HMBC correlations between H-3 to C-4; H_2_-9 and H_2_-10 to C-8 (carbonyl carbon); H-11 to C-13; H_2_-16 to C-1 and H_3_-17 to C-1, C-15 and C-16; H_3_-18 to C-3, C-4 and C-5; H_3_-19 to C-8 and C-9; and H_3_-20 to C-11, C-12 and C-13 permitted the connection of the carbon skeleton (Figure 1). Furthermore, the HMBC correlation observed from the methoxy protons (δ 3.34, 3H, s) to the carbon resonating at δ 105.6 positioned a methoxy group at C-7. In considering the degrees of unsaturation and molecular formula, two oxa-bridged ether linkages were placed between C-3/C-14 and C-4/C-7 by HMBC correlations from H-14 to C-3 and H-7 to C-4. The relative configuration of **5** was determined by the interpretation of the NOESY correlations (Figure 4). It was found that H_3_-18 showed NOE interactions with H-1, H-3 and methoxy protons (H_3_-21). Thus, by considering a molecular model as shown in Figure 4 and assuming the β-orientation of H_3_-18, all of H-1, H-3 and methoxy group should be positioned on the β face. The NOE correlation observed between H-1 and H-14 also reflected the β-orientation of H-14. Furthermore, NOESY spectrum showed NOE interaction of H_3_-20 with H-10, but not with H-11, revealing the *E* geometry of the C-11/C-12 double bond. From the above evidence and the other NOE correlations (Figure 4) the relative configurations at chiral centers of **5** was assumed to be 1R*, 3R*, 4R*, 7R* and 14S*. On the basis of the above analysis, the structure of **5** was established.

It is worth noting that metabolites **1**–**4** are rare cembranoids possessing a tetrahydrofuran moiety with a 3,14-ether linkage, which has been discovered previously in the soft coral *Sinularia gibberosa* [5,21]. In addition, **5** is the first secocembrane possessing two tetrahydrofuran moieties with 3,14- and 4,7-ether linkages. Our study thus adds the structure diversity of cembranoidal natural compounds.

The cytotoxicity of compounds **1**–**5** against the proliferation of a limited panel of cancer cell lines, including K562 (human chronic myelogenous leukemia), DLD-1 *(*human colon adenocarcinoma) and HepG2 and Hep3B (human liver carcinoma), was studied. The results showed that **1**–**5** are not cytotoxic toward the above cancer cells (IC_50_ > 20 μg/mL).

## 3. Experimental Section

### 3.1. General Experimental Procedures

The melting points were determined using a Fisher-Johns melting point apparatus. Optical rotation values were measured with a JASCO P-1010 digital polarimeter. IR spectra were recorded on a VARIAN DIGLAB FTS 1000 Fourier transform infrared spectrophotometer. The NMR spectra were recorded on a VARIAN MERCURY PLUS 400 FT-NMR (or Varian Unity INOVA 500 FT-NMR) instrument at 400 MHz (or 500 MHz) for ^1^H NMR and 100 MHz (or 125 MHz) for ^13^C NMR, respectively, in CDCl_3_. ESIMS were recorded on a Bruker APEX II mass spectrometer. Silica gel 60 (Merck, 230–400 mesh) was used for column chromatography. Precoated silica gel plates (Merck, Kieselgel 60 F254, 0.25 mm) and precoated RP-18 F254S plates (Merck, 1.05560) were used for TLC analysis. High-performance liquid chromatography (HPLC) was performed on a Hitachi L-7100 pump equipped with a Hitachi L-7400 UV detector at 210 nm. A semipreparative reversed-phase column (250 × 10 mm, 5 μm) and a preparative normal phase column (250 × 21.2 mm, 5 μm) was used for HPLC.

### 3.2. Animal Material

The soft coral *Lobophytum* sp. was collected by hand using SCUBA off the coast of Dongsha Atoll, in April, 2007, at a depth of 10 m, and stored in a freezer until extraction. A voucher specimen (Specimen No. DA2007-04-20) was deposited in the Department of Marine Biotechnology and Resources, National Sun Yat-sen University.

### 3.3. Extraction and Separation

The frozen soft coral (1.5 kg, fresh wt) was minced and extracted exhaustively with EtOAc (5 × 1 L). The organic extract was evaporated to yield a residue (21.9 g), which was fractionated by open column chromatography on silica gel using *n*-hexane–EtOAc and EtOAc–MeOH mixtures of increasing polarity to yield 16 fractions. Fraction 5, eluting with *n*-hexane–EtOAc (15:1), was further separated by silica gel column chromatography with gradient elution (*n*-hexane–EtOAc, 15:1 to 5:1) to yield five subfractions (5A–5E). Subfraction 5C was subjected to normal phase HPLC (*n*-hexane–EtOAc, 15:1) to obtain compound **2** (2.5 mg). Fractions 7 and 8, eluting with *n*-hexane–EtOAc (5:1), were combined and further separated over silica gel column chromatography (*n*-hexane–EtOAc, gradient elution, 5:1 to 1:1) to give four subfractions (7A–7D). Subfraction 7A was further purified by RP-18 HPLC (CH_3_CN–H_2_O, 3:2) to yield compound **5** (2.2 mg). In the same manner, compound **1** (4.2 mg) was obtained from subfraction 7B using RP-18 HPLC (CH_3_CN–H_2_O, 5:2). Fraction 11, eluting with *n*-hexane–EtOAc (1:1), was further separated by silica gel column chromatography with gradient elution (*n*-hexane–EtOAc, 1:1 to 1:5) to yield five subfractions (11A–11E). Subfraction 11C was further purified by RP-18 HPLC (CH_3_CN–H_2_O, 1:1) to yield compounds **3** (3.0 mg) and **4** (2.5 mg).

Lobophylin A (**1**): colorless oil; [α]*_D_*^25^ = −39 (*c* 0.3, CHCl_3_); IR (neat) ν_max_ 3460, 2926, 1649, 1458, 1381 and 1215 cm^−1; 1^H and ^13^C NMR data, see Tables 1 and 2; ESIMS *m*/*z* 343 [100, (M + Na)^+^]; HRESIMS *m*/*z* 343.2251 (calcd for C_20_H_32_O_3_Na, 343.2249).

Lobophylin B (**2**): colorless oil; [α]*_D_*^25^ = −35 (*c* 0.3, CHCl_3_); IR (neat) ν_max_ 3445, 2926, 1649, 1456, 1376 and 1265 cm^−1; 1^H and ^13^C NMR data, see Tables 1 and 2; ESIMS *m*/*z* 327 [100, (M + Na)^+^]; HRESIMS *m*/*z* 327.2301 (calcd for C_20_H_32_O_2_Na, 327.2300).

Lobophylin C (**3**): white powder; mp 76–78 °C; [α]*_D_*^25^ = +30 (*c* 0.1, CHCl_3_); IR (neat) ν_max_ 3377, 2927, 1649, 1459, 1377 and 1269 cm^−1; 1^H and ^13^C NMR data, see Tables 1 and 2; ESIMS *m*/*z* 343 [100, (M + Na)^+^]; HRESIMS *m*/*z* 343.2248 (calcd for C_20_H_32_O_3_Na, 343.2249).

Lobophylin D (**4**): white powder; mp 68–70 °C; [α]*_D_*^25^ = +22 (*c* 0.2, CHCl_3_); IR (neat) ν_max_ 3425, 2924, 1640, 1455, 1379 and 1240 cm^−1; 1^H and ^13^C NMR data, see Tables 1 and 2; ESIMS *m*/*z* 343 [100, (M + Na)^+^]; HRESIMS *m*/*z* 343.2246 (calcd for C_20_H_32_O_3_Na, 343.2249).

Lobophylin E (**5**): colorless oil; [α]*_D_*^25^ = +19 (*c* 0.2, CHCl_3_); IR (neat) ν_max_ 3444, 2929, 1715, 1640, 1454, 1374 and 1214 cm^−1; 1^H and ^13^C NMR data, see Tables 1 and 2; ESIMS *m*/*z* 373 [100, (M + Na)^+^]; HRESIMS *m*/*z* 373.2356 (calcd for C_21_H_34_O_4_Na, 373.2355).

### 3.4. Cytotoxicity Testing

Cell lines were purchased from the American Type Culture Collection (ATCC). Cytotoxicity assays of compounds **1**–**5** were performed using the MTT [3-(4,5-dimethylthiazol-2-yl)-2,5-diphenyltetrazolium bromide] colorimetric method [22].

### 3.5. Molecular Mechanics Calculations

Implementation of the MM2 force filed in Chem3D Pro software from Cambridge Soft Corporation, Cambridge, MA, USA (ver. 9.0, 2005), was used to calculate molecular models.

## Figures and Tables

**Table 1:** ^13^C NMR data for compounds **1**–**5**.

C#	1 a	2 a	3 a	4 b	5 b
1	50.2 (CH) c	49.0 (CH)	49.3 (CH)	49.3 (CH)	49.8 (CH)
2	29.1 (CH_2_)	27.4 (CH_2_)	26.7 (CH_2_)	26.7 (CH_2_)	30.9 (CH_2_)
3	77.5 (CH)	77.6 (CH)	77.8 (CH)	77.6 (CH)	82.2 (CH)
4	74.5 (C)	74.2 (C)	74.6 (C)	74.7 (C)	86.6 (C)
5	39.1 (CH_2_)	38.6 (CH_2_)	42.5 (CH_2_)	43.3 (CH_2_)	31.9 (CH_2_)
6	23.8 (CH_2_)	21.5 (CH_2_)	118.9 (CH)	121.8 (CH)	33.3 (CH_2_)
7	64.7 (CH)	126.6 (CH)	142.7 (CH)	141.5 (CH)	105.6 (CH)
8	60.3 (C)	132.8 (C)	73.6 (C)	72.6 (C)	208.9 (C)
9	38.1 (CH_2_)	38.2 (CH_2_)	44.4 (CH_2_)	43.7 (CH_2_)	43.7 (CH_2_)
10	23.9 (CH_2_)	24.4 (CH_2_)	23.5 (CH_2_)	22.2 (CH_2_)	22.5 (CH_2_)
11	126.5 (CH)	127.1 (CH)	129.4 (CH)	129.6 (CH)	124.2 (CH)
12	133.0 (C)	131.9 (C)	130.9 (C)	130.8 (C)	134.2 (C)
13	40.2 (CH_2_)	39.3 (CH_2_)	38.9 (CH_2_)	38.8 (CH_2_)	39.7 (CH_2_)
14	78.5 (CH)	76.7 (CH)	76.0 (CH)	76.0 (CH)	80.3 (CH)
15	141.6 (C)	142.4 (C)	142.2 (C)	142.3 (C)	144.0 (C)
16	111.3 (CH_2_)	111.0 (CH_2_)	111.2 (CH_2_)	111.1 (CH_2_)	112.2 (CH_2_)
17	25.0 (CH_3_)	23.5 (CH_3_)	23.5 (CH_3_)	23.5 (CH_3_)	22.5 (CH_3_)
18	24.6 (CH_3_)	23.1 (CH_3_)	21.6 (CH_3_)	21.9 (CH_3_)	24.2 (CH_3_)
19	19.8 (CH_3_)	16.3 (CH_3_)	29.6 (CH_3_)	28.3 (CH_3_)	29.9 (CH_3_)
20	17.3 (CH_3_)	15.4 (CH_3_)	15.4 (CH_3_)	15.5 (CH_3_)	16.5 (CH_3_)
OMe					54.3 (CH_3_)

a Spectra recorded at 100 MHz in CDCl_3_;

b Spectra recorded at 125 MHz in CDCl_3_;

c Attached protons were deduced by DEPT experiments.

**Table 2:** ^1^H NMR data for compounds **1**–**5**.

	1 a	2 a	3 a	4 b	5 b
1	2.77 dt (8.8, 8.0) c	2.73 dt (11.2, 7.2)	2.73 dt (8.0, 8.8)	2.74 dt (9.0, 8.5)	2.78 dt (7.5, 8.5)
2	2.16 m; 1.92 m	2.08 m; 1.90 m	2.04 m; 1.86 m	2.05 m; 1.86 m	1.96 m; 1.91 m
3	3.98 dd (9.6, 4.4)	3.97 dd (9.6, 4.5)	3.82 dd (10.0, 4.8)	3.82 dd (9.5, 4.5)	3.98 dd (7.5, 7.5)
5	1.97 m; 1.70 m	1.94 m; 1.53 m	2.40 dd (14.0, 10.0); 2.05 m	2.40 dd (14.0, 10.0); 2.10 m	2.40 dd (14.0, 10.0); 1.94 m
6	2.05 m; 1.31 m	2.25 m; 2.06 m	5.60 ddd (15.2, 10.0, 5.2)	5.51 ddd (15.5, 10.0, 5.0)	2.02 m; 1.94 m
7	3.27 d (6.8)	5.17 dd (6.0, 6.0)	5.70 d (15.6)	5.75 d (15.5)	5.00 d (4.5)
9	1.86 m; 1.52 m	2.14 m; 1.96 m	1.92 m; 1.58 m	1.95 m; 1.58 m	2.45 dd (8.0, 7.0)
10	2.21 m; 1.88 m	2.32 m; 2.04 m	2.19 m; 2.10 m	2.56 m; 1.96 m	2.27 dd (7.5, 7.5)
11	5.09 t (6.8)	4.89 d (8.0)	4.96 d (9.6)	4.94 d (10.0)	5.12 dd (7.0, 6.5)
13	1.95 m; 1.68 m	1.88 m; 1.72 m	1.91 m; 1.64 m	1.92 m; 1.64 m	2.00 m; 1.97 m
14	4.37 ddd (12.0, 3.6, 3.6)	4.36 ddd (11.6, 5.2, 4.8)	4.33 ddd (11.6, 6.0, 5.2)	4.33 ddd (12.0, 6.0, 5.5)	4.14 ddd (9.0, 4.5, 3.5)
16	4.87 d (1.6); 4.81 s	4.85 d (1.2); 4.78 s	4.86 d (1.6); 4.80 s	4.86 d (1.0); 4.80 s	4.83 s; 4.72 s
17	1.77 s	1.75 s	1.76 s	1.76 s	1.75 s
18	1.15 s	1.09 s	1.11 s	1.13 s	1.28 s
19	1.24 s	1.65 s	1.28 s	1.37 s	2.13 s
20	1.61 s	1.57 s	1.67 s	1.70 s	1.65 s
OMe					3.34 s

a Spectra recorded at 400 MHz in CDCl_3_;

b Spectra recorded at 500 MHz in CDCl_3_;

c *J* values (in Hz) in parentheses.
